# Management of the Pulpless Teeth

**Published:** 1898-10

**Authors:** J. Taft


### Management of the Pulpless Teeth.

BY J. TAFT.

Read before the Section in Stomatology, A. M. A., Denver, June, 1898.

Two thoughts induce me to present the following suggestions upon the subject indicated by the above title, namely, first, that pulpless teeth and roots are susceptible of being retained in the mouth and made serviceable and comfortable under a proper treatment, for a much longer period than is usually realized in the modes of treatment adopted.

Secondly, faulty treatment is so very common that it would seem important that some effort ought to be made for a better mode of proceedure in this particular of practice. It is entirely familiar to all dentists of much observation and discrimination that by far the larger share of disease and discomfort from affected teeth occur in cases where the pulps have been destroyed (usually the result of decay). Disease and severe pain many a time occurs before devitalization of the tooth pulp, and without some appropriate treatment the teeth are disintegrated and destroyed.

Physiologically teeth pulps were intended to serve a valuable purpose during the life of the organs with which they are associated. There is an important function which they should serve during this period, but unfortunately from one cause or another there are multitudes upon multitudes of pulpless teeth and roots.

The common occasion of death of this tissue is by decay of the teeth: subjecting the pulp to exposure, irritation, inflamation, hypertrophy, suppuration and death, this is the common result of this process.

Pulps are, however, frequently devitalized in other ways. calcific deposit in the pulp chamber or in the pulp itself or upon the apex of the root, cutting off its vascular and nerve supply, is not an infrequent occasion of pulpless teeth. In a low state of health when the nutrient function is impaired, the pulp sometimes becomes devitalized without any apparent cause. The conservation of exposed tooth pulp is a mode of practice that under favorable circumstances and with proper skill is entirely practicable; in a large percent of exposed pulps, as they are found, it is the result of decay. The indications presented in many cases are not such as to promise much good for conservative effort, and hence it occurs many times that the destruction of the pulp is a necessity if the best results are to be attained.

The best method of accomplishing this is not always employed. Many methods have been used which we will not fully consider here. It is hardly proper to pass from these points, however, without two or three suggestions. One is that when an exposed tooth pulp is to be devitalized and removed, its conditions should be as thoroughly understood as possible, and not only this, but the peculiarities, whether normal or abnormal, should be thoroughly understood. The practice of applying poisonous escharotics, as arsenious acid for example, should always be avoided. With the appliances and facilities at the command of every dentist, the use of such agents is wholly unnecessary, and especially so, when it is considered that most serious results follow their use in many instances, in either the immediate or remote future, to those highly susceptible of the poisonous influence of arsenious acid or similar agents. Specific results in some instances occur within twenty-four hours, these results, however, may be in other cases delayed for weeks or months before manifesting any action in a very marked manner.

It is better that these agents never be used for this purpose and that some methods should be employed that would not leave a sting behind. Since cocaine has been used as an anesthetic, there has been no occasion for using a more violent agent, as by proper application or injection hyperdomically or application to, or injection of the agent into the pulp, it may be so anesthetized that its painless removal is an operation entirely practicable.

Since the general introduction of anesthesia by cocaine and the electric current (cataphoresis) it is a very simple operation to render an exposed pulp entirely oblivious to contact, when it can as easily be removed from its habitat as any other tissue of the body.

This can be done by the use of the barbed broach or a sharp pulp extractor. In the performance of this operation the teeth should be perfectly protected by the rubber dam from the saliva or any agent that would convey infection.

The instruments employed in the operation should be absolutely aseptic, and kept as nearly so as possible during the operation.

The broaches, if they are used, should be moistened during the operation with some efficient antiseptic fluid. The instruments used in cleaning out the canal should be used with the same precaution.

The pulp chamber and canal that has been treated in this manner is in the best possible condition for permanent enclosure, unless there is a persistent hemorrhage or weeping of plasm from the foramen into the canal, and that, in most cases is readily arrested by some appropriate styptic or coagulant, as Lugol's solution, tannin, per-sulphate of iron and a number of other things that will suggest themselves to the intelligent dentist.

In by far the larger portion of cases this flow will cease spontaneously or in a very brief time, but if not, it may be assisted as above indicated.

When the pulp has been brought to this condition and thoroughly dried by a jet of hot air or by the use of a copper root dryer, it is in the best possible condition for permanent filling. In such cases nothing can be gained by the very commonly prolonged treatment preparatory to filling. The best time for closing such cases is immediately. If there is doubt as to the entire arrest of the plasm flow through the root, it might have packed into the canal, a small pledget of lint moistened with some astringent, then fill the canal with hill stopping or some material that will be impervious. Twenty-four hours will be sufficient to determine whether the flow has been entirely arrested; before removing the temporary stopping the rubber dam should again be used, when it will be determined whether there remains any discharge, if not, the cavity may be wiped with lint, moistened with a solution of salicylic acid, when the canal and pulp chamber should at once be thoroughly filled with a material that will wholly exclude moisture in any form. Care should be exercised in filling the extreme part of the canal where there is no disease in the apical space.

Any treatment after the canal and pulp chamber has been brought into the condition above described is liable to be fraught with disaster. It is a very common practice with many to persist in what is denominated "root treatment" for days, weeks and sometimes months, in just such cases as this; a practice that ought to be discarded and condemned.

In filling roots of teeth many methods are employed, the aim in every case should be to absolutely close the extreme end of the canal and the canal itself, in such a manner as to prevent the entrance of septic matter, or anything that would convey it.

Roots of teeth have been filled with lead, tin foil, gold foil, gold wire, gutta percha in solution and solid, hill stopping in solution and solid. By any of these methods in skillful hands this work can be very perfectly and efficiently accomplished.

The late Dr. Wm. N. Morrison, of St. Louis, practiced filling roots with gold wire made as nearly the size and shape of the canal to be filled as could be estimated, shaping the wire so that it would pass to the end of the canal, then covered the wire with a solution of gutta percha or hill stopping and force it to its position in the canal, cutting it off in the pulp chamber. The same method was adopted when lead was used instead of gold, by the late Prof. Peabody, of Louisville, Ky. Shaping the lead so as to fit as closely into the canal as possible, covering the lead with a gutta percha solution, press or drive it to its place in the canal, and cut it off in the pulp chamber.

Dr. H. J. McKellops follows practically the same method, using however, a cone of gutta percha or hill stopping the size and shape of the canal, moistened with a solution and pressing it to its place. These methods or modifications are much used in filling roots of teeth.

Dr. C. R. Butler, of Cleveland, O., fills roots of teeth usually with tin foil, cutting into narrow strips, packing into the canal with an appropriate filling instrument, by this means absolutely sealing it.

Gold foil used in the same way has no advantage over tin.

The conditions of root management as above indicated are the most simple and the most successful. In the preparation of decayed teeth with open pulp chambers the tissue has a number of phases. The forms more simple than those described above are those cases where the pulp from exposure has become diseased and devitalized as the result, and is simply sluffed away or remains a lifeless mass in the canal, with little or no discharge from the foramen at the end of the root, and no periostial disturbance.

In these cases the requirements are, first to wash out the cavity as thoroughly as possible with a fine syringe with an antiseptic fluid, then with an appropriate instrument remove all debris that is not washed away, then open and shape the canal so as to readily facilitate the work to be done. Disinfection and antisepsis is always required more or less for these cases. It is well in some cases to remove more of the dentine from the walls of the canal than would be required in those from which a living pulp had recently been removed. This is necessary because the decomposing matter in the canal enters the canals and vitiates their contents to a greater or less extent, according to the susceptibility of this material and the offensive condition of the contents of the pulp canal. The contents of the canals of the dentine in many cases remarkably resist the pernicious influence of the fetid matter of the pulp canal, but in other cases is greatly susceptible to its pernicious influences.

In the latter case it is important that the walls of the canal be cut away so as to remove this condition of the dentine. In such a case a thorough antiseptic and disinfectant treatment should be employed. In many cases much more should be done in the way of cutting from the canal walls than is done by many. In this case sulphuric acid may be employed with very decided benefit. It readily enters the open canal and dissolves the dentine, is an antiseptic, and would arrest decomposition of the contents of the dentine canals, so far as it may be brought in contact with them.

Care should be exercised in the use of sulphuric acid for this purpose, that it does not pass through the foramen at the end of the root, as it would there be an active irritant.

After the canal has been prepared in the manner suggested, it is important to determine whether there is any discharge of any sort from the apical foramen into the canal, if so, that condition must be changed. This can in some instances be effectually done by thoroughly filling the extreme end of the canal. This proceedure is practicable in cases where the flow is not copious and consists in the main of the plasm of the blood. This arrest may in some cases be promptly accomplished by the use of a co-agulant packed well into the canal and permitted to remain for a few days.

This method of treatment is usually efficient where there is but little discharge and this of a limpid and inoffensive character and with a good constitution. In many cases, however, the discharge is so abundant that neither sealing nor the use of the coagulant will be immediately effective, but either or both together may be used, checking the flow in part at least until the disposition to discharge is relieved. This discharge may come from a remnant of pulp in the extreme part of the canal, or in the absence of this, it may come from the irritated living tissue just outside the apical foramen. This treatment in many cases, especially those of a more favorable kind, will be effective in arresting the discharge in twenty-four hours. Whenever this has been accomplished the canal may be promptly and permanently filled.

From the pulp canals of many teeth, however, the discharge is acrid and proves a source of irritation if promptly arrested or suddenly checked. In such cases disinfectant or antiseptic treatment may be used in connection with partial stopping, thus rendering the discharge less offensive and less irritating to the living tissue beyond.

Pyrozone three percent solution may be used in such cases daily with decided benefit. After this treatment, tannin and oil of cassia may be used with linen or cotton firber, and placed tightly in the extreme part of the canal.

Solution of salicylic acid may also be used with very decided benefit. If a remnant of pulp is found in the canal it should always be removed before the treatment suggested. It is oftentimes the case that the sudden arrest of discharge will prove an active irritant to the tooth in the apical space and more or less pronouncedly to the entire surrounding membrane of the root. That should in all cases where possible be avoided by the proper regulation of the treatment employed. When the discharge in such cases is arrested the canal and cavity should be properly and thoroughly filled.

I have thus far presented only the most favorable cases in the management of pulpless teeth, emphasizing only the following points:

First—When the tooth pulp is to be destroyed it is important to avoid any irritating or poisonous agents for this purpose; anesthesia of course is in most cases required, this is readily accomplished by injection of cocaine or by cataphoresis. In a good many cases neither of these are required, but it is insisted that all poisonous agents should be avoided.

Second—The removal of debris and offensive matter from the tooth that is to be conserved should be most thorough. The utmost cleanliness and purity should be observed in every step of the work. The fluids of the mouth should be effectually excluded.

Third—Excavation in the canal is made for two purposes.

One to enlarge and make it more accessible in every part of the work. The other is rendered necessary where there is decomposition taking place, especially in the contents of the dentine canals. In many instances all this offensive material may in this way be removed and thus the danger of further trouble lessened.

Fourth—The importance of arrest in every variety of discharge that may be taking place from the pulp canal; and let it be borne in mind that this usually does not involve a prolonged treatment. There are many cases that have had extended treatment, the results of which are disease and oftentimes loss of the tooth.

Under favorable circumstances nature always makes rapid work in the reparation of diseased tissue. I have, however, purposely omitted the management of pulpless teeth that have involved the surrounding tissue, those in which abcesses have been formed and in which there may be diseased gums of one variety or another.

These conditions may form a basis of consideration at another time.

